# Markov Chain Realization of Multiple Detection Joint Integrated Probabilistic Data Association

**DOI:** 10.3390/s19010112

**Published:** 2018-12-30

**Authors:** Yuan Huang, Taek Lyul Song, Dae Hoon Cheagal

**Affiliations:** 1 Department of Electronic Systems Engineering, Hanyang University, Ansan 15588, Korea; hy4335657@hotmail.com; 2 LIG System, Seoul 03130, Korea; dhcheagal@ligcorp.com

**Keywords:** Markov chain process, multiple detection, target existence evaluation, multitarget tracking, data association

## Abstract

In multiple detection target tracking environments, PDA-based algorithms such as multiple detection joint integrated probabilistic data association (MD-JIPDA) utilize the measurement partition method to generate measurement cells. Thus, one-to-many track-to-measurements associations can be realized. However, in this structure, the number of joint data association events grows exponentially with the number of measurement cells and the number of tracks. MD-JIPDA is plagued by large increases in computational complexity when targets are closely spaced or move cross each other, especially in multiple detection scenarios. Here, the multiple detection Markov chain joint integrated probabilistic data association (MD-MC-JIPDA) is proposed, in which a Markov chain is used to generate random data association sequences. These sequences are substitutes for the association events. The Markov chain process significantly reduces the computational cost since only a few association sequences are generated while keeping preferable tracking performance. Finally, MD-MC-JIPDA is experimentally validated to demonstrate its effectiveness compared with some of the existing multiple detection data association algorithms.

## 1. Introduction

Target tracking and information fusion techniques have achieved more attention in recent years due to their wide applications in both military and civilian domains [1,2,3,4,5,6]. In multitarget tracking environments, the data association process decides which selected measurement comes from which target and evaluates the corresponding association probability [7,8,9]. Usually, a target can be detected once with a less-than-unity detection probability, and false alarms (clutter) are also present in the surveillance area, which leads to more challenges for the data association process.

Tracks, which are formed to estimate the trajectories of the targets, are initialized using measurements; however, they are initialized without prior information of the measurement origins. This means that true tracks that are following targets and false tracks that are following clutter are both initialized and that they propagate during the surveillance period. The problem of true and false track discrimination is introduced, known as the false track discrimination (FTD) problem in [7,10].

Among the various tracking approaches, multiple hypothesis tracking (MHT) is an algorithm that utilizes multiple-scan track-to-measurement association by evaluating the likelihoods of the association hypotheses as specified in [11] and Chapter 6.3 of [12]. In MHT, hypotheses, which can be viewed as the measurement resource declarations at each scan, are generated and updated, and then the hypothesis with the highest a posteriori probability is the resulting output for track acceptance and rejection at each scan. As we know, MHT has issues with its computational complexity, in which the number of hypotheses grows exponentially. Some heuristics have been proposed to relax the complexity [11,13,14], but there is nevertheless a sacrifice in optimality.

The joint integrated probabilistic data association (JIPDA) algorithm [8] is a pseudo-Bayesian estimator that enumerates all track-to-measurement associations and calculates the corresponding weights. JIPDA is a single-scan algorithm that implements associations between the current scan tracks and the selected measurements. Instead of trying to find one “best” measurement for a track, all measurements selected by the track are evaluated and a track state is generated by the summation of the state corresponding to each data association event over all the weighted association events. In order to obtain the association weights, the summation of the data association probabilities over all association events is needed, which is an NP-hard problem [15,16]. It has been proved that JIPDA is much more efficient compared to MHT for closely spaced targets and dense clutter environments, resulting in the extensive applicability of JIPDA.

Since JIPDA suffers from a heavy computational load, a suboptimal method is proposed in [17], called linear multitarget integrated probabilistic data association (LM-IPDA). In this algorithm, after track *t* selects various measurements, the measurement generated by the target being tracked by another track is treated as additional clutter for track *t*. This additional clutter is used to modulate the origin clutter measurement density, which allows LM-IPDA to totally bypass the joint data association step. This clutter modification process is the core of the LM approach, which upgrades the single target tracking algorithm to a multitarget tracking algorithm. This algorithm reduces the complexity of heavy multiple target tracking to that of single target tracking, but sacrifices optimality in the process.

In MHT, the hypothesis with the highest probability is utilized to accept and reject tracks, and PDA-based algorithms calculate the consecutive detection probability of each track in order to terminate unstable tracks [2,11,12,18]. In [7,19], the probability of target existence (PTE) is introduced as a track score, which is continuously updated (along with the track state) and used to confirm the track (i.e., the target tracked by the corresponding track exists). The PTE of each track is updated considering the ratios of measurement likelihood to clutter measurement density for all of the measurements selected by that track. Compared to MHT, which uses a global hypothesis, each track has a PTE, allowing JIPDA to perform track judgment for each track separately. Compared to the consecutive detection probability used by JPDA, PTE has a more stable performance.

JIPDA enumerates all possible association events in order to approximate the optimal Bayesian filter, which suffers from a large computational complexity, especially when targets are closely spaced. The Markov chain JIPDA (MC-JIPDA) generates the association events via a Markov chain process [20]. In each event generation step for a track, the current track-to-measurement assignment is only correlated with the last assignment and independent of the other tracks. The main benefit of this approach is that the number of association sequences can be controlled and only a small number of association sequences are needed. One drawback is that, repeat association sequences can be generated in the MC-JIPDA algorithm, as all association events are generated randomly.

Due to the applications of high resolution sensors and some special kinds of radars such as over-the-horizon-radar (OTHR), multiple detection target tracking generally attracts more attention from the research community [9,21,22,23,24,25,26]. For such multiple detection situations, the widely used point target assumption is relaxed and the data association process needs to assign multiple measurements to one track, which leads to the association complexity exponentially increasing compared to the single detection case.

The measurement partition method [21] is used to generate the measurement cells for each track, where each cell is a combination of selected measurements that are assumed to be target detections. This method is a mathematical technique that can be smoothly incorporated into any existing tracking algorithms. However, the number of measurement cells quickly increases with an increasing number of selected measurements, which results in an extremely high computational complexity at the track-to-measurement cell association step. Since multiple detection JIPDA (MD-JIPDA) enumerates all possible association events, it is not feasible in many multiple detection applications due to the computational resources that are required [27]. Multiple detection LM-IPDA (MD-LM-IPDA) is efficient in these multiple detection scenarios, but afflicts the deteriorating tracking performance [28].

The contributions: The multiple detection Markov chain joint integrated probabilistic data association (MD-MC-JIPDA) algorithm is proposed to solve the multiple detection target tracking problem based on a much more efficient data association sequence generation process. Instead of enumerating all feasible joint events (FJEs) for data associations among measurement cells and tracks, MD-MC-JIPDA generates a certain number of FJEs based on the Markov chain sequence of each track. Then, the corresponding event probabilities are evaluated using the measurement cells and track states under consideration. The track state and probability of target existence are updated based on these FJEs. The main benefit of this algorithm is that it needs only a small number of FJEs and this number is decided in advance and can be adjusted according to the complexity of the tracking scenario. The novel FJEs generation mechanism makes MD-MC-JIPDA algorithm much more efficient and tractable in multiple detection multitarget tracking environments.

This paper is organized as follows. The assumptions and models are described in Section 2. The structure of MD-MC-JIPDA is demonstrated in Section 3. The simulation studies and conclusions are given in Section 4 and Section 5, respectively.

## 2. Assumptions and Models

This section provides the details of the assumptions and models used in this paper. Targets usually occur and disappear at random times and can be detected with a less-than-unity probability [18]. Targets become even harder to detect if they maneuver in certain ways [29]. In the bearing only case, in order to track targets, the sensor needs to navigate with more complex maneuvers compared to the targets in order to satisfy the observability condition [30].

### 2.1. Target Motion

The most widely used nearly constant velocity (NCV) model, in Chapter 4.2 of [31], is considered here, where the target state evolves according to

(1) $$x_{k+1}^{t}=Ax_{k}^{t}+v_{k}^{t},$$

where $x_{k}^{t}$ is the state of target *t* at scan *k*, *A* is the state propagation matrix, and $v_{k}^{t}$ represents the zero-mean white Gaussian process noise with covariance *Q*.

### 2.2. Measurements

The standard multiple detection situation, which is caused by a high resolution sensor that can resolve multiple scattering feature points of a target, is considered. A target can be detected $\varphi_{t}$ times with the corresponding given detection probability $P_{D\varphi_{t}}$. Target measurements are generated by

(2) $$\begin{array}{c} \begin{array}{c} z_{k}=H_{\varphi_{t}}x_{k}^{t}+w_{\varphi_{t}}\left(k\right), \end{array} \end{array}$$

where the parameters $H_{\varphi_{t}}$ and $\omega_{\varphi_{t}}$ are given by

(3) $$\begin{array}{c} H_{\varphi_{t}}=\underset{\varphi_{t}}{⊕}H \end{array}$$

(4) $$\begin{array}{c} \begin{array}{c} w_{\varphi_{t}}\left(k\right)=\underset{\varphi_{t}}{⊕}w\left(k\right) \end{array} \end{array}$$

in which $H=\left[1,0\right]⊗I_{2}$ is the measurement generation matrix for a single detection and the sign ⊕ represents the vertical vectorial concatenation operation. $w\left(k\right)$ is the Gaussian measurement noise that $p\left(w\left(k\right)\right)=N\left(w\left(k\right);0,R\right)$ in which *R* is the sensor error covariance. $\varphi_{t}$ used here represents the number of target detections such that $H_{\varphi_{t}}$ and $w_{\varphi_{t}}\left(k\right)$ correspond to the case that there are $\varphi_{t}$ detections from target *t* at scan *k*.

False alarms (clutter measurements) also arise in the surveillance area. This kind of measurement is assumed to follow the Poisson/uniform distribution in this paper.

The set of measurements selected at scan *k* is represented by $Z_{k}$, which contains both target measurements and clutter measurements, given by

(5) $$Z_{k}={\left\{z_{k,j}\right\}}_{j=1}^{m_{k}},$$

where $z_{k,j}$ represents the *j*th measurement and $m_{k}$ is the total number of selected measurements at scan *k*.

The set of sets of measurements collected from the initial to current scan is $Z^{k}$, which satisfies

(6) $$Z^{k}=\left\{Z_{1},Z_{2},\ldots ,Z_{k}\right\}.$$

At each scan, the measurements selected by a track are used to estimate the target state and to evaluate the target existence probability under the multiple detection paradigm.

## 3. Multiple Detection Markov Chain Joint Integrated Probabilistic Data Association

This section demonstrates the detailed derivations of MD-MC-JIPDA. We first introduce the track state and the measurement partition method and then focus on the structure for jointly assigning measurement cells to tracks. The contribution of MD-MC-JIPDA algorithm lies in the efficient joint assignment mechanism.

When the targets are closely spaced or move across each other, the computational burden of the joint association events increases sharply, hampering the implementation of the traditional tracking algorithms such as MHT and JIPDA. Furthermore, the multiple detection situation significantly aggravates this burden since the number of measurement cells of each track is usually much larger compared to the number of measurements selected by that track. Therefore, in an attempt to realize a real-time algorithm, the multiple detection version of the Markov chain process is proposed as an approximation of the Bayes estimator.

### 3.1. Track State

For a detector, there is no a priori information on the measurement origins, resulting in that a track may track a target or clutter. Thus, the existence of the target being tracked by a track is a random event. The probability of this random event is termed the probability of target existence $P\left(\chi_{k}^{t}|Z^{k}\right)$. In MD-MC-JIPDA the track state pdf is represented by

(7) $$p\left[x_{k}^{t},\chi_{k}^{t}|Z^{k}\right]=p\left(x_{k}^{t}|\chi_{k}^{t},Z^{k}\right)P\left(\chi_{k}^{t}|Z^{k}\right)$$

which consists of the trajectory state and the target existence event. On the RHS of (7), we can see that the kinematic state $x_{k}^{t}$ is conditional on the target existence $\chi_{k}^{t}$. Both $p\left(x_{k}^{t}|\chi_{k}^{t},Z^{k}\right)$ and $P\left(\chi_{k}^{t}|Z^{k}\right)$ are propagated according to a standard predict-update mechanism [7,8].

### 3.2. Measurement Utilization

At each scan, each track uses the gating method, which can be found in Chapter 2.3.2 of [2], to select measurements. Since the multiple detection problem is considered, the measurements selected by a track are first used to generate measurement cells. Then, the measurement cells are used for the data association in order to update the PTE and the state of the corresponding track. Assume that track *t* selects three measurements $\left\{z_{k,1},z_{k,2},z_{k,3}\right\}$ and the maximum number of target originated measurements $\varphi_{t,\max}$ is 3. Then, the measurement cells are generated as follows:

$$\begin{array}{c} \begin{array}{c} \left\{\left\{z_{k,1}\right\},\left\{z_{k,2}\right\},\left\{z_{k,3}\right\}\right\} \end{array} \end{array}$$

where $z_{1,1}\left(k\right)=\left\{z_{k,1}\right\}$, $z_{1,2}\left(k\right)=\left\{z_{k,2}\right\}$ and $z_{1,3}\left(k\right)=\left\{z_{k,3}\right\}$. In this case $\varphi_{t}=1$, $c_{1}=C_{1}^{3}=3$ and $n_{1}\in \left\{1,2,3\right\}$.

$$\begin{array}{c} \begin{array}{c} \left\{\left\{z_{k,1},z_{k,2}\right\},\left\{z_{k,1},z_{k,3}\right\},\left\{z_{k,2},z_{k,3}\right\}\right\} \end{array} \end{array}$$

where $z_{2,1}\left(k\right)=\left\{z_{k,1},z_{k,2}\right\}$, $z_{2,2}\left(k\right)=\left\{z_{k,1},z_{k,3}\right\}$ and $z_{2,3}\left(k\right)=\left\{z_{k,2},z_{k,3}\right\}$. In this case $\varphi_{t}=2$, $c_{2}=C_{2}^{3}=3$ and $n_{2}\in \left\{1,2,3\right\}$.

$$\begin{array}{c} \begin{array}{c} \left\{\left\{z_{k,1},z_{k,2},z_{k,3}\right\}\right\} \end{array} \end{array}$$

where $z_{3,1}\left(k\right)=\left\{z_{k,1},z_{k,2},z_{k,3}\right\}$. In this case $\varphi_{t}=3$, $c_{3}=C_{3}^{3}=1$ and $n_{3}\in \left\{1\right\}$.

Then, these measurement cells are used in the joint data association process instead of using the single measurements $z_{k,1}$, $z_{k,2}$ and $z_{k,3}$.

### 3.3. Feasible Joint Event

In this part, we give a brief review of the feasible joint events of MD-JIPDA and introduce a new perspective on the probability of a feasible joint event, preparing for the derivation of MD-MC-JIPDA.

Under the multiple detection condition, measurement cells, which are composed of one or more selected measurements, are assigned to tracks in a feasible joint event [2]. In the following derivations, we assume that the cluster tracks can select all the measurements in the cluster to form feasible joint events [2].

In MD-JIPDA, the feasible joint events are used to generate the track-to-measurement cell assignments. In each feasible joint event, the assignments for all the cluster tracks and all the measurement cells are considered. The probability of a feasible joint event $\varepsilon_{j}$ in MD-JIPDA is calculated by

(8) $$\begin{array}{c} P\left(\varepsilon_{j}|Z^{k}\right)=\kappa^{-1}\prod_{t\in T_{0}^{\varepsilon_{j}}}\left[1-P_{Dec}^{t}P\left(\chi_{k}^{t}|Z^{k-1}\right)\right]\cdot \prod_{t\in T^{\varepsilon_{j}}}\left[P_{DG\varphi_{t}}P\left(\chi_{k}^{t}|Z^{k-1}\right)\frac{p_{z_{\varphi_{t},n_{\varphi_{t}}}}}{\rho^{\varphi_{t}}}\varphi_{t}!\right], \end{array}$$

where each track is assigned one measurement cell or is unassigned, and any two measurement cells assigned to different tracks do not share common measurements [27].

The truncated measurement cell likelihood $p_{z_{\varphi_{t},n_{\varphi_{t}}}}$ in (8) for $z_{\varphi_{t},n_{\varphi_{t}}}\left(k\right)$ is calculated by

(9) pzφt,nφt=Nz⌢φt,nφtk;z¯φt,nφtk,SkNz⌢φt,nφt;z¯φt,nφt,SkPGφtPGφt.

where ${\overset{⌢}{\;z}}_{\varphi_{t},n_{\varphi_{t}}}\left(k\right)$ is the concatenated measurement based on measurement cell $z_{\varphi_{t},n_{\varphi_{t}}}\left(k\right)$, ${\bar{z}}_{\varphi_{t},n_{\varphi_{t}}}\left(k\right)$ is the predicted measurement and $S_{k}$ represents the corresponding innovation covariance. The details for obtaining these parameters can be referred to [28].

In (8), $P_{Dec}^{t}$ is the probability that at least one target measurement is detected and is located in the selection gate of track *t*, given as

(10) $$P_{Dec}^{t}=\sum_{\varphi_{t}=1}^{\varphi_{t,\max}}P_{DG\varphi_{t}},$$

where $P_{DG\varphi_{t}}$ is the probability that $\varphi_{t}$ measurements are detected and are located in the selection gate of track *t*, given by

(11) $$P_{DG\varphi_{t}}=P_{D\varphi_{t}}{\left(P_{G}\right)}^{\varphi_{t}},$$

and ρ is the clutter measurement density.

The predicted probability of target existence is given by

(12) $$P\left(\chi_{k}^{t}|Z^{k-1}\right)=p_{11}P\left(\chi_{k-1}^{t}|Z^{k-1}\right),$$

where $p_{11}$ is the transition probability that a target exists at the previous scan and keeps its existence state at the current scan, which is usually set as 0.98 [7].

The normalization constant κ used in (8) can be obtained based on the fact that the total probability of all data association events is

(13) $$\sum_{j=1}^{M}P\left(\varepsilon_{j}|Z^{k}\right)=1,$$

where *M* is the number of joint events.

In all the derivations, $p_{z_{\varphi_{t},n_{\varphi_{t}}}}$ is used as an abbreviation of the measurement cell likelihood $p\left(z_{\varphi_{t},n_{\varphi_{t}}}\left(k\right)|\chi_{k}^{t},Z^{k-1}\right)$ and this value is calculated by a modulated Kalman filter which will be given later.

Let us have another look at each joint event in terms of the tracks. Define $\eta_{\varepsilon_{j}}^{t}=z_{\varphi_{t},n_{\varphi_{t}}}\left(k\right)$ as the event that measurement cell $z_{\varphi_{t},n_{\varphi_{t}}}\left(k\right)$ is assigned to track *t* under joint event $\varepsilon_{j}$, and $\eta_{\varepsilon_{j}}^{t}=z_{0}\left(k\right)$ as the event that there is no measurement assigned to track *t* under joint event $\varepsilon_{j}$. The corresponding probabilities can be found in (8) as

(14) $$\begin{array}{c} P\left(\eta_{\varepsilon_{j}}^{t}=z_{\varphi_{t},n_{\varphi_{t}}}\left(k\right)\right)=P_{DG\varphi_{t}}P\left(\chi_{k}^{t}|Z^{k-1}\right)\frac{p_{z_{\varphi_{t},n_{\varphi_{t}}}}}{\rho^{\varphi_{t}}}\varphi_{t}! \end{array}$$

and

(15) $$P\left(\eta_{\varepsilon_{j}}^{t}=z_{0}\left(k\right)\right)=1-P_{Dec}^{t}P\left(\chi_{k}^{t}|Z^{k-1}\right).$$

The probability of a feasible joint event $\varepsilon_{j}$, from the point of view of tracks $\varepsilon_{j}=\left\{\eta_{\varepsilon_{j}}^{1},\ldots ,\eta_{\varepsilon_{j}}^{N}\right\}$, can be rewritten as

(16) $$P\left(\varepsilon_{j}|Z^{k}\right)=\kappa^{-1}\prod_{t=1}^{N}P\left(\eta_{\varepsilon_{j}}^{t}\right).$$

where *N* is the total number of tracks.

Therefore, a feasible joint event consists of the measurement cell-to-track assignments for all the cluster tracks, in which each track is assigned with a measurement cell ($z_{\varphi_{t},n_{\varphi_{t}}}\left(k\right)$ or $z_{0}\left(k\right)$).

### 3.4. Markov Chain Sequence

In MD-JIPDA, the number of feasible joint events grows exponentially with the number of measurement cells and the number of tracks involved. When MD-JIPDA is used for closely spaced multitarget tracking considering clutter measurements, the computational load for the feasible joint event probability calculation becomes intractable. This is the fatal weakness for applying MD-JIPDA or any other algorithms which use feasible joint events for data association to real-time multiple detection environments. Therefore, the algorithm with a limited number feasible joint events should be executed for real-time applications. In addition, the limited size feasible joint events need to represent the significant joint events and neglect insignificant joint events to obtain a reasonable data association performance.

Let us consider the Markov process which can be used to sequentially assign measurement cell to a track. The Markov process satisfies

(17) $$\begin{array}{c} P\left(\eta_{n+1}=a_{n+1}|\eta_{n}=a_{n},\eta_{n-1}=a_{n-1},\ldots ,\eta_{1}=a_{1}\right)=P\left(\eta_{n+1}=a_{n+1}|\eta_{n}=a_{n}\right), \end{array}$$

which indicates that the state at current time $\eta_{n+1}$ depends only on the last state $\eta_{n}$ and has nothing to do with the previous states. Utilizing the property of (17), one can generate the state transition much more efficiently since not the entire past state but only the last state is necessary for the current state generation.

Utilizing the Markov property in (17), we can sequentially generate Markov chain. For the measurement cell-to-track assignment process, a Markov chain can be represented by the corresponding matrix $\Delta^{t}$ of which each element $\Delta_{ef}^{t}$ is the transition probability from selecting $z_{e}\left(k\right)$ to selecting $z_{f}\left(k\right)$. The transition probabilities for each track are defined as

(18) $$\Delta_{ef}^{t}\overset{\Delta }{\;=}\Delta \left(\eta_{\varepsilon_{j+1}}^{t}=z_{f}\left(k\right)|\eta_{\varepsilon_{j}}^{t}=z_{e}\left(k\right)\right),$$

which represents that $z_{e}\left(k\right)$ is assigned to track *t* under joint event $\varepsilon_{j}$ and $z_{f}\left(k\right)$ is assigned to track *t* under $\varepsilon_{j+1}$, where $e,f\in \left\{0,\left(\varphi_{t},n_{\varphi_{t}}\right)\right\}$. These transition probabilities satisfy

(19) $$\sum_{f}\Delta_{ef}^{t}=1.$$

The transition probabilities that satisfy the condition that the current selection is the same as the last one are

(20) $$\begin{array}{cc} \Delta_{ee}^{t}= & \frac{P_{DG\varphi_{t}}P\left(\chi_{k}^{t}|Z^{k-1}\right)p_{z_{\varphi_{t},n_{\varphi_{t}}}}\varphi_{t}!}{\kappa \rho^{\varphi_{t}}},\;\;\;\;\;e=\left(\varphi_{t},n_{\varphi_{t}}\right) \end{array}$$

and

(21) $$\Delta_{ee}^{t}=\frac{1-P_{Dec}^{t}P\left(\chi_{k}^{t}|Z^{k-1}\right)}{\kappa },\;\;\;\;\;e=0.$$

where these values are generated according to (14) and (15).

Assume that the number of measurement cells of track *t* at scan *k* is $M_{c}^{t}\left(k\right)$. The transition probabilities that satisfy the condition that the current selection is different from the last one are given by

(22) $$\begin{array}{cc} \Delta_{ef}^{t}= & \frac{1}{M_{c}^{t}\left(k\right)}\left[1-P_{DG\varphi_{t}}P\left(\chi_{k}^{t}|Z^{k-1}\right)\cdot \frac{p_{z_{\varphi_{t},n_{\varphi_{t}}}}}{\kappa \cdot \rho^{\varphi_{t}}}\varphi_{t}!\right],\;\;e=\left(\varphi_{t},n_{\varphi_{t}}\right),e\neq f \end{array}$$

and

(23) $$\begin{array}{c} \Delta_{ef}^{t}=\frac{1}{M_{c}^{t}\left(k\right)}\left[1-\right.\frac{1-P_{Dec}^{t}P\left(\chi_{k}^{t}|Z^{k-1}\right)}{\kappa }],\;\;e=0,e\neq f. \end{array}$$

The normalization constant κ of these transition probabilities is given as

(24) $$\begin{array}{c} \kappa =1-P_{Dec}^{t}P\left(\chi_{k}^{t}|Z^{k-1}\right)+\sum_{\varphi_{t}}\sum_{n_{\varphi_{t}}}P_{DG\varphi_{t}}P\left(\chi_{k}^{t}|Z^{k-1}\right)\frac{p_{z_{\varphi_{t},n_{\varphi_{t}}}}}{\rho^{\varphi_{t}}}\varphi_{t}!. \end{array}$$

In each feasible joint event, any two measurement cells assigned to different tracks should not contain the same measurements.

The transition probability matrix for each track is given as (25). This matrix considers all possible transitions among the measurement cells (including $z_{0}\left(k\right)$) of a track.

(25) $$\begin{array}{c} \begin{array}{cc} \Delta^{t}= & \left[\begin{array}{cccc} \Delta_{0,0}^{t} & \Delta_{0,\left(1,1\right)}^{t} & \Delta_{0,\left(1,2\right)}^{t} & \Delta_{0,\left(2,1\right)}^{t}\\ \Delta_{\left(1,1\right),0}^{t} & \Delta_{\left(1,1\right),\left(1,1\right)}^{t} & \Delta_{\left(1,1\right),\left(1,2\right)}^{t} & \Delta_{\left(1,1\right),\left(2,1\right)}^{t}\\ \Delta_{\left(1,2\right),0}^{t} & \Delta_{\left(1,2\right),\left(1,1\right)}^{t} & \Delta_{\left(1,2\right),\left(1,2\right)}^{t} & \Delta_{\left(1,2\right),\left(2,1\right)}^{t}\\ \Delta_{\left(2,1\right),0}^{t} & \Delta_{\left(2,1\right),\left(1,1\right)}^{t} & \Delta_{\left(2,1\right),\left(1,2\right)}^{t} & \Delta_{\left(2,1\right),\left(2,1\right)}^{t} \end{array}\right]\\ = & \left[\begin{array}{cccc} \frac{1-P_{Dec}^{t}P\left(\chi_{k}^{t}|Z^{k-1}\right)}{\kappa } & \frac{1}{3}\left(1-\Delta_{0,0}^{t}\right) & \frac{1}{3}\left(1-\Delta_{0,0}^{t}\right) & \frac{1}{3}\left(1-\Delta_{0,0}^{t}\right)\\ \frac{1}{3}\left(1-\Delta_{\left(1,1\right),\left(1,1\right)}^{t}\right) & \frac{P_{DG1}P\left(\chi_{k}^{t}|Z^{k-1}\right)p_{z_{1,1}}}{\kappa \rho } & \frac{1}{3}\left(1-\Delta_{\left(1,1\right),\left(1,1\right)}^{t}\right) & \frac{1}{3}\left(1-\Delta_{\left(1,1\right),\left(1,1\right)}^{t}\right)\\ \frac{1}{3}\left(1-\Delta_{\left(1,2\right),\left(1,2\right)}^{t}\right) & \frac{1}{3}\left(1-\Delta_{\left(1,2\right),\left(1,2\right)}^{t}\right) & \frac{P_{DG1}P\left(\chi_{k}^{t}|Z^{k-1}\right)p_{z_{1,2}}}{\kappa \rho } & \frac{1}{3}\left(1-\Delta_{\left(1,2\right),\left(1,2\right)}^{t}\right)\\ \frac{1}{3}\left(1-\Delta_{\left(2,1\right),\left(2,1\right)}^{t}\right) & \frac{1}{3}\left(1-\Delta_{\left(2,1\right),\left(2,1\right)}^{t}\right) & \frac{1}{3}\left(1-\Delta_{\left(2,1\right),\left(2,1\right)}^{t}\right) & \frac{2P_{DG2}P\left(\chi_{k}^{t}|Z^{k-1}\right)p_{z_{2,1}}}{\kappa \rho^{2}} \end{array}\right] \end{array} \end{array}$$

Suppose two tracks, *t* and t+1, have selected the same measurements $\left\{z_{k,1},z_{k,2}\right\}$ and $\varphi_{t,\max}=\varphi_{t+1,\max}=2$. After measurement cell generation process, three measurement cells are generated, which are $z_{1,1}\left(k\right)=\left\{z_{k,1}\right\}$, $z_{1,2}\left(k\right)=\left\{z_{k,2}\right\}$ and $z_{2,1}\left(k\right)=\left\{z_{k,1},z_{k,2}\right\}$, for both track *t* and t+1. The Markov chain state set is $\left\{z_{0}\left(k\right),z_{1,1}\left(k\right),z_{1,2}\left(k\right),z_{2,1}\left(k\right)\right\}$. The state transition for a track, such as *t*, from $\eta_{\varepsilon_{j}}^{t}=z_{e}\left(k\right)$ to $\eta_{\varepsilon_{j+1}}^{t}=z_{f}\left(k\right)$ is accepted with probability $\Delta_{ef}^{t}$.

#### 3.4.1. Data Association Sequences for a Track

**Figure 1:**
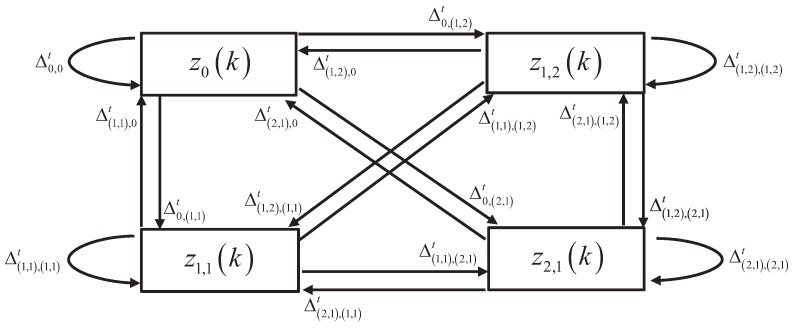
Transition relationship among the measurement cells of a track.

An example of the transition relation among measurement cells of track *t* is shown in Figure 1, in which $z_{0}\left(k\right)$, $z_{1,1}\left(k\right)$, $z_{1,2}\left(k\right)$ and $z_{2,1}\left(k\right)$ are considered. From this figure, each measurement cell can transform to the other measurement cells with corresponding transition probabilities. Suppose that track *t* selects $z_{1,2}\left(k\right)$ in the data association sequence $\eta_{\varepsilon_{j}}^{t}$, which means

(26) $$\eta_{\varepsilon_{j}}^{t}=z_{1,2}\left(k\right);$$

then the third row of (25) should be used to determine which measurement cell should be selected for track *t* in the next data association sequence $\eta_{\varepsilon_{j+1}}^{t}$. Assume the corresponding transition probabilities are

(27) $$\begin{array}{cc} \Delta_{\left(1,2\right),0}^{t}=0.2, & \Delta_{\left(1,2\right),\left(1,1\right)}^{t}=0.2 \end{array}$$

and

(28) $$\begin{array}{cc} \Delta_{\left(1,2\right),\left(1,2\right)}^{t}=0.4, & \Delta_{\left(1,2\right),\left(2,1\right)}^{t}=0.2. \end{array}$$

Then generate a random probability $P\in \left[0,1\right]$ to select a measurement cell for $\eta_{\varepsilon_{j+1}}^{t}$ based on (29). Suppose that P=0.35, which indicates that $z_{1,1}\left(k\right)$ should be chosen for track *t* in the data association sequence $\eta_{\varepsilon_{j+1}}^{t}$.

(29) $$\begin{array}{c} 0\leq P\leq \Delta_{\left(1,2\right),0}^{t},\;\;\;\eta_{\varepsilon_{j+1}}^{t}=z_{0}\left(k\right)\\ \Delta_{\left(1,2\right),0}^{t}<P\leq \Delta_{\left(1,2\right),0}^{t}+\Delta_{\left(1,2\right),\left(1,1\right)}^{t},\;\;\;\eta_{\varepsilon_{j+1}}^{t}=z_{1,1}\left(k\right)\\ \Delta_{\left(1,2\right),0}^{t}+\Delta_{\left(1,2\right),\left(1,1\right)}^{t}<P\leq \Delta_{\left(1,2\right),0}^{t}+\Delta_{\left(1,2\right),\left(1,1\right)}^{t}+\Delta_{\left(1,2\right),\left(1,2\right)}^{t},\;\;\;\eta_{\varepsilon_{j+1}}^{t}=z_{1,2}\left(k\right)\\ \Delta_{\left(1,2\right),0}^{t}+\Delta_{\left(1,2\right),\left(1,1\right)}^{t}+\Delta_{\left(1,2\right),\left(1,2\right)}^{t}<P\leq \Delta_{\left(1,2\right),0}^{t}+\Delta_{\left(1,2\right),\left(1,1\right)}^{t}+\Delta_{\left(1,2\right),\left(1,2\right)}^{t}+\Delta_{\left(1,2\right),\left(2,1\right)}^{t},\;\;\;\eta_{\varepsilon_{j+1}}^{t}=z_{2,1}\left(k\right) \end{array}$$

The measurement cell selection for track *t* in $\eta_{\varepsilon_{j+1}}^{t}$ is only related to the selection of track *t* in $\eta_{\varepsilon_{j}}^{t}$ based on the transition matrix, which is the core of the proposed Markov chain sequences. Based on this process, track *t* generates the Markov chain sequence of length *K* which is ${\left\{\eta_{\varepsilon_{j}}^{t}\right\}}_{j=1}^{K}$, and then track t+1 also generates its Markov chain sequence of length *K* following the same procedure.

#### 3.4.2. Joint Data Association Events for Multiple Tracks

If $\eta_{\varepsilon_{j+1}}^{t+1}=z_{e}\left(k\right)\neq z_{0}\left(k\right)$ and $\eta_{\varepsilon_{j+1}}^{t}=z_{f}\left(k\right)\neq z_{0}\left(k\right)$, and $z_{e}\left(k\right)$ and $z_{f}\left(k\right)$ contain the same measurement, then regenerate $\eta_{\varepsilon_{j+1}}^{t+1}$ until it selects the measurement cell which has no common measurement with $z_{f}\left(k\right)$ to satisfy the condition of the multiple detection feasible joint event. Using the transform relation given in Figure 1 and the length of Markov chain sequence *K* is set to be 5, i.e., the number of FJEs in MD-MD-JIPDA is 5. The possible Markov chain sequence for track *t* and t+1 can be ${\left\{\eta_{\varepsilon_{j}}^{t}\right\}}_{j=1}^{5}=\left\{z_{0}\left(k\right),z_{1,1}\left(k\right),z_{0}\left(k\right),z_{2,1}\left(k\right),z_{1,2}\left(k\right)\right\}$ and ${\left\{\eta_{\varepsilon_{j}}^{t+1}\right\}}_{j=1}^{5}=\left\{z_{2,1}\left(k\right),z_{1,2}\left(k\right),z_{1,1}\left(k\right),z_{0}\left(k\right),z_{2,1}\left(k\right)\right\}$. Then, we need to check whether the track-to-measurement cell association sequences denoted by $\left\{\eta_{\varepsilon_{j}}^{t},\eta_{\varepsilon_{j}}^{t+1}\right\},\;\;\;j=1,\ldots ,5$ satisfy the multiple detection feasible joint event condition.

**Figure 2:**
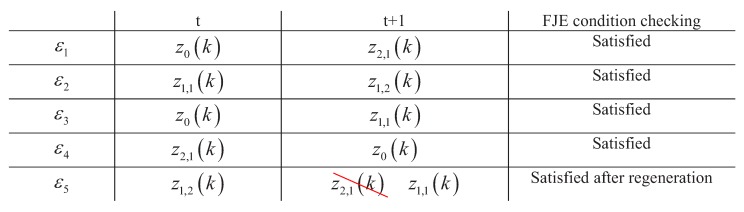
Feasible joint event generation in multiple detection Markov chain joint integrated probabilistic data association (MD-MC-JIPDA).

Figure 2 demonstrates the feasible joint events generation process using the Markov chain association sequences of track *t* and t+1.

In this example, $\left\{\eta_{\varepsilon_{1}}^{t},\eta_{\varepsilon_{1}}^{t+1}\right\}=\left\{z_{0}\left(k\right),z_{2,1}\left(k\right)\right\}$, $\left\{\eta_{\varepsilon_{2}}^{t},\eta_{\varepsilon_{2}}^{t+1}\right\}=\left\{z_{1,1}\left(k\right),z_{1,2}\left(k\right)\right\}$, $\left\{\eta_{\varepsilon_{3}}^{t},\eta_{\varepsilon_{3}}^{t+1}\right\}=\left\{z_{0}\left(k\right),z_{1,1}\left(k\right)\right\}$, $\left\{\eta_{\varepsilon_{4}}^{t},\eta_{\varepsilon_{4}}^{t+1}\right\}=\left\{z_{2,1}\left(k\right),z_{0}\left(k\right)\right\}$ and $\left\{\eta_{\varepsilon_{5}}^{t},\eta_{\varepsilon_{5}}^{t+1}\right\}=\left\{z_{1,2}\left(k\right),z_{2,1}\left(k\right)\right\}$. Among them $\left\{\eta_{\varepsilon_{5}}^{t},\eta_{\varepsilon_{5}}^{t+1}\right\}=\left\{z_{1,2}\left(k\right),z_{2,1}\left(k\right)\right\}$ violates the multiple detection feasible event condition since $z_{1,2}\left(k\right)$ and $z_{2,1}\left(k\right)$ contain the same measurement $z_{k,2}$. So, $\eta_{\varepsilon_{5}}^{t+1}$ of track t+1 should be regenerated until the multiple detection feasible joint event condition is satisfied.

Then, the probability for the feasible joint event $\varepsilon_{j}=\left\{\eta_{\varepsilon_{j}}^{t},\eta_{\varepsilon_{j}}^{t+1}\right\},\;\;\;j=1,\ldots ,5$ is obtained by (16). The length of total feasible joint events *K* in MD-MC-JIPDA can be predetermined based on the complexities of different scenarios.

### 3.5. Track Update

The association probabilities of a measurement cell to a track are generated based on the corresponding feasible joint events. For simplicity, the time index *k* in $z_{\varphi_{t},n_{\varphi_{t}}}\left(k\right)$ and $z_{0}\left(k\right)$ is omitted. Denote by $\Xi \left(t,z_{\varphi_{t},n_{\varphi_{t}}}\left(k\right)\right)$ the set of feasible joint events that allocate cell $z_{\varphi_{t},n_{\varphi_{t}}}\left(k\right)$ to track *t*. Notice that if there is no feasible joint event that allocates measurement cell $z_{\varphi_{t},n_{\varphi_{t}}}\left(k\right)$ to track *t*, the association probability for this measurement cell is 0.

The event that no measurement in the cluster is target *t* detection is the union of the data association sequences that allocate $z_{0}\left(k\right)$ to track *t*, given by

(30) $$P\left(\eta^{t}=z_{0}\left(k\right)\right)=\sum_{\varepsilon_{j}\in \Xi \left(t,z_{0}\left(k\right)\right)}P\left(\varepsilon_{j}|Z^{k}\right).$$

The probability that no measurement in the cluster comes from target *t* and that target *t* exists is expressed as

(31) $$\begin{array}{c} \begin{array}{c} P\left(\eta^{t}=z_{0}\left(k\right),\chi_{k}^{t}|Z^{k}\right)=\frac{\left(1-P_{Dec}^{t}\right)p\left(\chi_{k}^{t}|Z^{k-1}\right)}{1-P_{Dec}^{t}p\left(\chi_{k}^{t}|Z^{k-1}\right)}P\left(\eta^{t}=z_{0}\left(k\right)|Z^{k}\right). \end{array} \end{array}$$

The probability that measurement cell $z_{\varphi_{t},n_{\varphi_{t}}}\left(k\right)$ originates from target *t* and that target *t* exists is

(32) $$P\left(\eta^{t}=z_{\varphi_{t},n_{\varphi_{t}}}\left(k\right),\chi_{k}^{t}|Z^{k}\right)=\sum_{\varepsilon_{j}\in \Xi \left(t,z_{\varphi_{t},n_{\varphi_{t}}}\left(k\right)\right)}P\left(\varepsilon_{j}|Z^{k}\right).$$

Events $\left\{\eta^{t},\chi_{k}^{t}\right\}$ are mutually exclusive and the union of these events is the target existence event $\chi_{k}^{t}$. Therefore, the a posteriori probability of target existence is calculated by

(33) $$\begin{array}{c} \begin{array}{c} P\left(\chi_{k}^{t}|Z^{k}\right)=P\left(\eta^{t}=z_{0}\left(k\right),\chi_{k}^{t}|Z^{k}\right)+\sum_{\varphi_{t}=1}^{\varphi_{t,\max}}\sum_{n_{\varphi_{t}}=1}^{c_{\varphi_{t}}}P\left(\eta^{t}=z_{\varphi_{t},n_{\varphi_{t}}}\left(k\right),\chi_{k}^{t}|Z^{k}\right). \end{array} \end{array}$$

The association probabilities are expressed by

(34) $$\begin{array}{c} \begin{array}{c} \beta_{k}^{t}\left(0\right)=p\left(\eta^{t}=z_{0}\left(k\right)|\chi_{k}^{t},Z^{k}\right)=\frac{P\left(\eta^{t}=z_{0}\left(k\right),\chi_{k}^{t}|Z^{k}\right)}{P\left(\chi_{k}^{t}|Z^{k}\right)} \end{array} \end{array}$$

and

(35) $$\begin{array}{c} \begin{array}{c} \beta_{k}^{t}\left(z_{\varphi_{t},n_{\varphi_{t}}}\left(k\right)\right)=p\left(\eta^{t}=z_{\varphi_{t},n_{\varphi_{t}}}\left(k\right)|\chi_{k}^{t},Z^{k}\right)=\frac{P\left(\eta^{t}=z_{\varphi_{t},n_{\varphi_{t}}}\left(k\right),\chi_{k}^{t}|Z^{k}\right)}{P\left(\chi_{k}^{t}|Z^{k}\right)}. \end{array} \end{array}$$

For each association event, there is an update state generated by the modulated Kalman filter using the corresponding measurement cell. The detailed process of track state update can be found in [28].

After obtaining the data association probabilities and corresponding update states, the state of track *t* is generated according to a Gaussian mixture that considers all the association events. The final output for each track contains a track state and the probability of target existence.

### 3.6. Computational Complexity Analysis

In this section, we analyze the complexity of MD-JIPDA and MD-MC-JIPDA.

Suppose that there are *N* cluster tracks and *M* measurement cells which do not contain the same measurement in the cluster, then the number of feasible joint events is obtained as [17] $M!N!\sum_{i=0}^{N}\frac{1}{i!\left(M-i\right)!\left(N-i\right)!}$ which has the complexity of $O\left(M^{N}\right)$ if M>N, or similarly the feasible joint event generation shows the complexity of $O\left(N^{M}\right)$ if N>M. From this, the number of feasible joint events increases exponentially with *M* and *N*.

Compared to MD-JIPDA, MD-MC-JIPDA is much more efficient when many tracks share measurements since MD-MC-JIPDA requires only a certain number of FJEs. The complexity of the joint measurement cell-to-track assignment of MD-MC-JIPDA is $O\left(1\right)$ since the number of the joint assignments required by MD-MC-JIPDA is a predetermined constant *K* which can be adjusted according to the complexity of the tracking scenario.

## 4. Simulation

**Figure 3:**
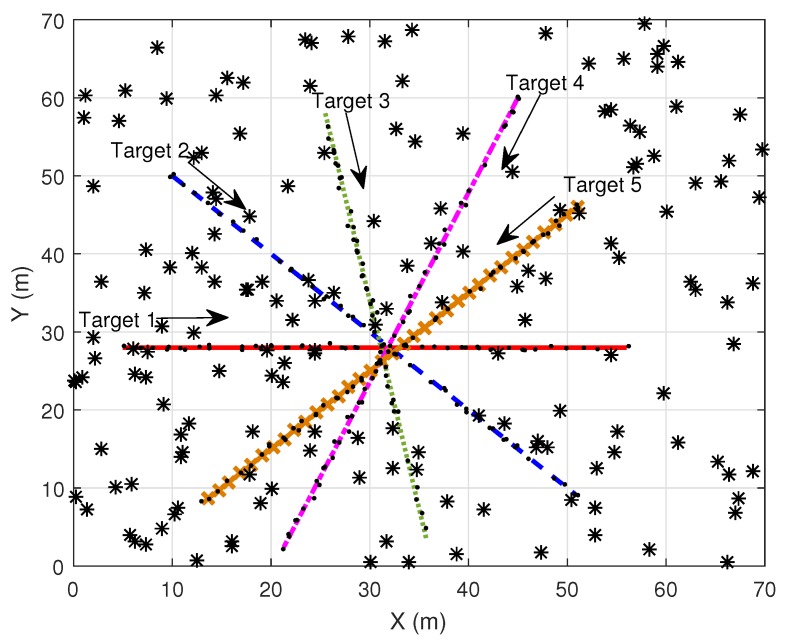
Simulation scenario (dots are target-originated measurements and asterisks are clutter measurements).

This section demonstrates the simulation performances of MD-LM-IPDA, MD-LM-ITS [32], MD-JIPDA and MD-MC-JIPDA in terms of target existence estimation, target state estimation accuracy, computational efficiency and OSPA distance [33,34]. As shown in Figure 3, five targets move in a $\left[0\;m,70\;m\right]\times \left[0\;m,70\;m\right]$ Cartesian coordinate using a NCV model. The target state is a four dimensional vector given by $\left[\begin{array}{cccc} x & y & \dot{x} & \dot{y} \end{array}\right]$, where *x* and *y* are the positions and $\dot{x}$ and $\dot{y}$ represent the velocities in the *X* and *Y* direction, respectively. The surveillance duration *T* is set to be 1 s. The state propagation and measurement generation equations are introduced in Section 2, in (1) and (2)

(36) $$A=\left[\begin{array}{cccc} 1 & 0 & T & 0\\ 0 & 1 & 0 & T\\ 0 & 0 & 1 & 0\\ 0 & 0 & 0 & 1 \end{array}\right],$$

(37) $$H=\left[\begin{array}{cccc} 1 & 0 & 0 & 0\\ 0 & 1 & 0 & 0 \end{array}\right],$$

and the covariance of $v_{k}$ is

(38) $$Q=q\cdot kron\left(\left[T^{3}/3,T^{2}/2;T^{2}/2,T\right],I_{2}\right),$$

where ‘*kron*’ represents the Kronecker product and $I_{2}$ is the two-by-two identity matrix. Note that the covariance of $w\left(k\right)$ is

(39) $$R=diag\left({\sigma_{x}}^{2},{\sigma_{y}}^{2}\right).$$

in which $\sigma_{x}=\sigma_{y}=0.5$ m.

There are many metrics that can influence the multitarget tracking performance such as (1). the clutter measurement density; (2). the target detection probability; and (3). the spacing of the targets. Tracking becomes more difficult when the targets are closely spaced and move across each other, which could result in ambiguity of the data association among tracks and measurements. Hence, these five targets move across each other around scan 19 to test the performance.

The two-point differencing, Chapter 3.2 in [2], is used to initialize tracks. At each scan, each track uses the gating method to select measurements. Once the measurement is selected, it is marked and will not be used for track initialization. The PTE is used to cover the false track discrimination problem and once the PTE of a track exceeds the confirmation threshold, it becomes a confirmed track and stays confirmed. Then, the following method is used to determine whether this confirmed track is a confirmed true track or a confirmed false track. 

(40) $$\begin{array}{c} condition\;\;\;1:{\left({\hat{x}}_{k|k}-x_{k}\right)}^{T}P_{0}^{-1}\left({\hat{x}}_{k|k}-x_{k}\right)\leq 20\\ condition\;\;\;2:{\left({\hat{x}}_{k|k}-x_{k}\right)}^{T}P_{0}^{-1}\left({\hat{x}}_{k|k}-x_{k}\right)\geq 40 \end{array}$$

Once track becomes a confirmed track, the normalized distance squared ${\left({\hat{x}}_{k|k}-x_{k}\right)}^{T}P_{0}^{-1}\left({\hat{x}}_{k|k}-x_{k}\right)$ is calculated. If this normalized distance squared is within the confirmed true track test threshold (≤20), the track becomes a confirmed true track for the corresponding target; if this normalized distance squared is out of the confirmed true track test threshold (>20), the track is a confirmed false track for the corresponding target. If the normalized distance squared of a confirmed true track exceeds the confirmed false track test threshold, which is set as 40 in this manuscript, this confirmed true track is counted as a confirmed false track for the corresponding target. Otherwise, it keeps the confirmed true track status for the corresponding target. At each scan, this normalized distance squared is calculated between each of the confirmed tracks and each of the targets. If there are many confirmed true tracks for one target or there are targets sharing the same confirmed true tracks, the auction algorithm [2] is used for the assignments between confirmed true tracks and targets. If a track is counted as the confirmed false track for all the targets, it is a confirmed false track, otherwise it is the confirmed true track. In (40), ${\hat{x}}_{k|k}$ is the state estimate at scan *k*, $x_{k}$ is the true target state at scan *k*, and $P_{0}$ represents the initial track covariance given by

(41) $$P_{0}=\left[\begin{array}{cc} R & R/T\\ R/T & 2R/T^{2} \end{array}\right].$$

When the track is initialized, it is assigned an initial PTE. The initial PTEs of MD-LM-IPDA, MD-LM-ITS, MD-JIPDA and MD-MC-JIPDA are different; this is so that these algorithms can be compared under the condition that all of them have the same number of confirmed false tracks. The values for the simulation parameters are shown in Table 1, where CFTs stands for the number of confirmed false tracks.

In order to obtain stable performances, data from 200 Monte Carlo simulation runs was used, where the surveillance period lasts 35 s. Only one sensor is located at the origin of the Cartesian coordinates which detects each target with probabilities $P_{D1}=0.5$ (the probability that there is a single target detection is 0.5) and $P_{D2}=0.4$ (the probability that there are two target detections is 0.4) at each scan. The amount of clutter at each scan follows a Poisson distribution with an average value of 5. The number of FJEs in MD-MC-JIPDA is set to be 300.

Here we introduce some parameters for track retention statistics and these parameters are counted before and after the target crossing:

- nCases: the number of tracks that are following a target at scan 13.
- nOK: the percentage of “nCases” tracks that are still following the original target at scan 33.
- nSwitched: the percentage of “nCases” tracks that end up following a different target at scan 33.
- nMerged: the percentage of “nCases” tracks that disappeared due to tracks merging between scan 13 and 33.
- nLost: the percentage of “nCases” tracks that are not following any target at scan 33.
- nResult: the number of tracks that are following a target at scan 35.
- CPU time [s]: the average computation time for one recursion cycle on a 3.10 GHz Intel PC platform and run with the Matlab Program.

These statistics are used to indicate the tracking performances before and after the target crossing. nCases is used to record the number of the confirmed true tracks at a certain time before the target crossing. nOK indicates the number of the confirmed true tracks that continuously track the same target before and after the target crossing. nSwitch indicates the number of tracks which swap the target after the target crossing. This happens from the influence of target measurement that is shared among cluster tracks and results in the tracking object changes without track termination. nMerged shows that after the target crossing, several tracks pursue the same target and thus they are merged due to similar target state estimates. nLost track is generated due to track errors, which results in the PTE drop below a certain threshold and the track is terminated. This kind of track loss usually results from the fact that the data association is invalid to some extent due to the target crossing. If nOK is bigger, it indicates that the tracking performance is better. The number of nOK tracks plus the number of nSwitch tracks comprise the number of the survived tracks in nCases tracks after the target crossing. The sum of the number of nMerged tracks and the number of nLost tracks becomes the number of terminated tracks. Finally, nResult shows the number of the confirmed true tracks at the end of the whole tracking period after the target crossing. These parameters together constitute the performance description of target tracks before and after the target crossing, which are important indices to verify the algorithm. The similar tracking performance analysis using these statistic parameters can be found in [5,28,35].

**Figure 4:**
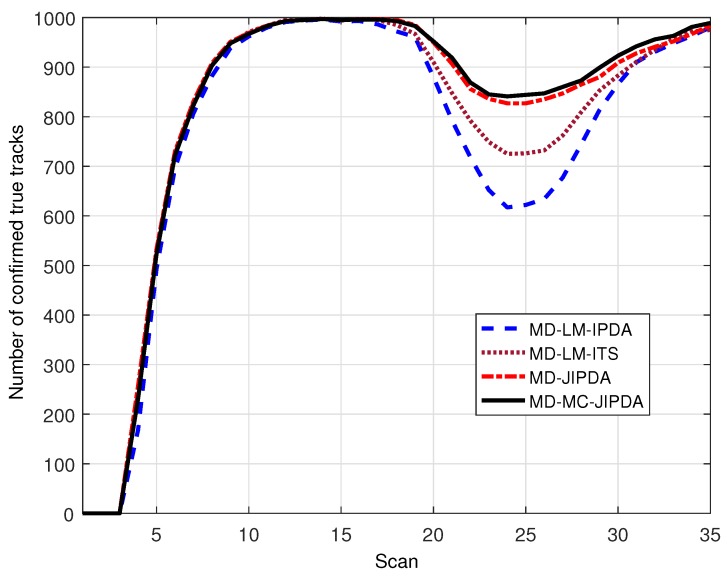
Number of confirmed true tracks for all targets.

**Figure 5:**
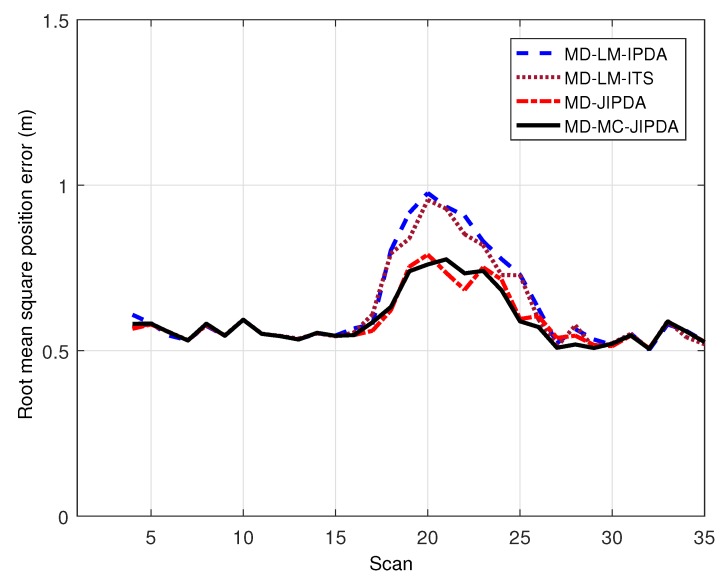
Root mean square error for target 5.

The number of confirmed true tracks for all five targets and the root mean square position error of target 5 are shown in Figure 4 and Figure 5, respectively. In Figure 4, the perfect number of confirmed true tracks (i.e., 100%) is 1000. There is a severe drop in the number of confirmed true track near the target crossing time, which indicates that all the algorithms in comparison experience data association difficulty when targets are located closely. However, when the targets intersect each other, there are obvious differences among the four algorithms, which indicates that MD-MC-JIPDA maintains many more tracks compared to LM-based algorithms and slightly more tracks compared to MD-JIPDA.

As for the root mean square position error, the performances of these four algorithms have the same trend of increases in the error when the targets cross. However, MD-JIPDA and MD-MC-JIPDA have obviously smaller position estimation errors compared to LM-based algorithms, which indicates MD-JIPDA and MD-MC-JIPDA are less affected by multitarget crossing. The increasing error near the target crossing leads to more shared measurements among tracks. From these results, one can see that MD-MC-JIPDA has the highest track retention rate with the satisfactory target state estimation accuracy compared with the other algorithms.

Table 2 demonstrates the track retention performances of MD-LM-IPDA, MD-LM-ITS, MD-JIPDA and MD-MC-JIPDA. From this table, MD-JIPDA and MD-MC-JIPDA are shown to have much higher percentages of nOK compared to the LM-based algorithms. MD-LM-ITS has better nOK performance compared to MD-LM-IPDA since the tracks in MD-LM-ITS maintain several track components, each component has a multi-scan data association history, for propagation, which makes MD-LM-ITS tracks more stable in the target crossing. Detailed analyzing for MD-LM-ITS is referred to [32]. MD-MC-JIPDA has a higher summation of nOK and nSwitched, which indicates more survived target tracks, and this is the reason that the CTT performance of MC-MC-JIPDA is much better compared to LM-based algorithms and slightly better compared to MD-JIPDA. Comparing the summation of nMerged and nLost, MD-MC-JIPDA has the lowest percentage of the terminated tracks. All these four algorithms have similar numbers of nResult, which suggests that the tracks are recovered after a certain time period by the track initialization.

By comparing the simulation times in Table 2, in which CPU time is the average execution time per each run, in seconds, one can see that MD-LM-IPDA, MD-LM-ITS and MD-MC-JIPDA require only a fraction of the CPU time needed for MD-JIPDA. MD-MC-JIPDA is an effective algorithm that can be processed in real-time for this scenario.

OSPA was used recently for multi-target tracking performance evaluation [33,34]. Here, we add the OSPA performance of these four algorithms for comparison. At each scan, the algorithm output the tracks with PTE higher than the threshold (given as 0.5) to generate the OSPA distance and cardinality. The other parameters used for these four algorithms are given in Table 1.

**Figure 6:**
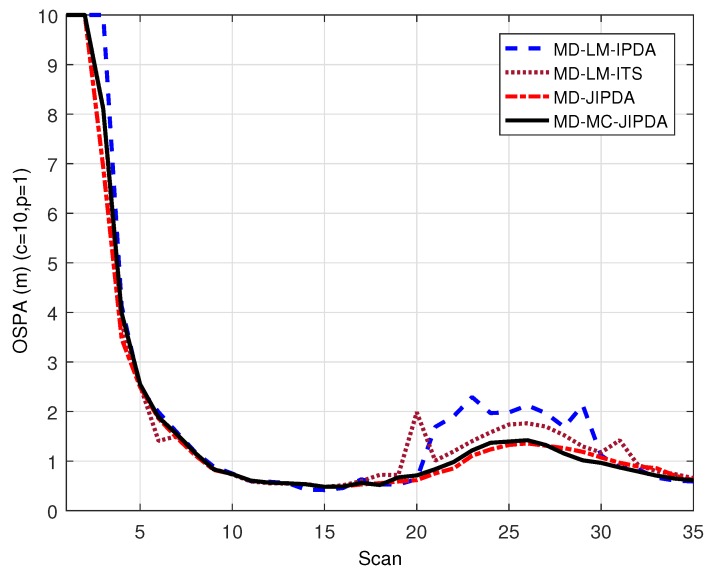
OSPA distance.

In Figure 6, OSPA distances (for *p* = 1 and *c* = 10) versus scan for 200 Monte Carlo simulation runs are shown. It can be seen that all these four algorithms show the same trend that OSPA distance is increased after the target crossing. The result suggests that both MD-JIPDA and MD-MC-JIPDA outperform MD-LM-ITS which in turn outperforms MD-LM-IPDA. Combined with the performance and the analysis given before, this result is due to the fact that MD-JIPDA and MD-MC-JIPDA have better data association performances when the cluster tracks share the cluster measurements.

**Figure 7:**
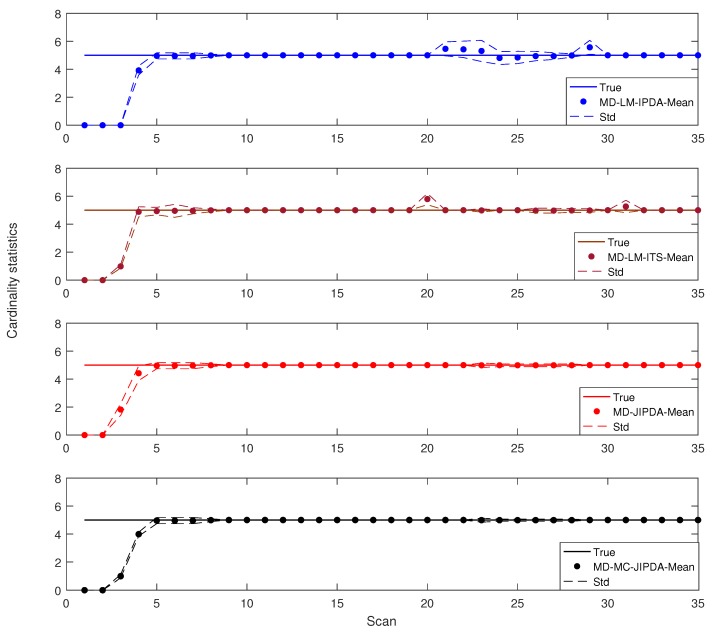
Cardinality statistics.

The cardinality statistics of these four algorithms are shown in Figure 7. From this figure one can see that MD-LM-IPDA has the worst tracking performance for the target crossing. The difference in cardinality statistics between MD-JIPDA and MD-MC-JIPDA is marginal. However, it can also be seen that MD-JIPDA and MD-MC-JIPDA have more reliable target number estimation performances.

## 5. Conclusions

The MD-MC-JIPDA algorithm is proposed for multiple detection multitarget tracking. Instead of enumerating all possible data association events, MD-MC-JIPDA generates a small number of feasible joint events according to the Markov chain sequences implemented by each of the cluster tracks. This joint data association mechanism significantly simplifies data association complexity.

In the scenario with a fixed number of targets crossing each other, MD-MC-JIPDA outperforms MD-LM-IPDA and MD-LM-ITS in the sense of the true track maintenance and the target trajectory estimation accuracy. MD-MC-JIPDA needs only a fraction of the simulation time required by MD-JIPDA but has a similar tracking performance compared with MD-JIPDA. From the tracking performance and the required simulation time, it can be seen that MD-MC-JIPDA is a real-time algorithm suitable for the multiple detection multitarget tracking.

The potential future works for the proposed algorithm are: (1) find the method to adaptively select the number of FJEs for MD-MC-JIPDA instead of predetermination; (2) in some scenarios, the switch of the tracks may cause the problem for the tracking consistency which encourages us to find a way to reduce the percentage of the track switch; (3) apply this tracking algorithm to the OTHR application. (3) ’fit’ the discrete estimates obtained by MD-MC-JIPDA to a continuous-time tracking function, which can be used to refine the estimates for any time in the effective fitting period [36]. 

## Figures and Tables

**Table 1:** Simulation parameters for different algorithms.

	MD-LM-IPDA	MD-LM-ITS	MD-JIPDA	MD-MC-JIPDA
Initial PTE	0.000025	0.000065	0.0001	0.000075
Confirmation PTE	0.95	0.95	0.95	0.95
Termination PTE	0.000025/2	0.000065/2	0.0001/2	0.000075/2
Number of CFTs	2	2	2	2

**Table 2:** Statistic parameters.

	MD-LM-IPDA	MD-LM-ITS	MD-JIPDA	MD-MC-JIPDA
nCases	994	995	995	995
nOK	43.13%	54.25%	68.84%	63.50%
nSwitched	18.14%	17.81%	13.97%	21.03%
nMerged	38.43%	27.84%	16.88%	14.57%
nLost	0.30 %	0.10%	0.31%	0.90%
nResult	980	975	984	989
CPU time [s]	0.42	2.49	202.97	1.69

## Abbreviations

The following abbreviations are used in this manuscript:

PDA	Probabilistic data association.
MD-JPDA	Multiple detection joint probabilistic data association.
MD-MC-JIPDA	Multiple detection Markov chain joint integrated probabilistic data association.
FTD	False track discrimination.
MHT	Multiple hypothesis tracker.
MAP	Maximum a posteriori.
JIPDA	Joint integrated probabilistic data association.
LM-IPDA	Linear multitarget integrated probabilistic data association.
PTE	Probability of target existence.
MC-JIPDA	Markov chain JIPDA.
OTHR	Over-the-horizon-radar.
MD-JIPDA	Multiple detection JIPDA.
MD-LM-IPDA	Multiple detection LM-IPDA.
MD-LM-ITS	Multiple detection linear multitarget integrated track splitting.
FJEs	Feasible joint events.
NCV	Nearly constant velocity.
CFT	Confirmed false track.
RMSE	Root mean square error.

## *Nomenclature*

*t*	A track as well as the potential target being tracked by this track.
$m_{k}$	The number of selected measurements at scan *k*.
*L*	The maximum number of scattering feature points of the target.
$\varphi_{t,\max}$	The maximum number of target-originated measurements, which satisfies $\varphi_{t,\max}=\min\left(L,m_{k}\right)$.
$\varphi_{t}$	The number of target originated measurements $\varphi_{t}\in \left\{1,2,\ldots ,\varphi_{t,\max}\right\}$.
$n_{\varphi_{t}}$	A variable that enumerates the measurement cells under the condition that there are $\varphi_{t}$ measurements generated by target *t*, $n_{\varphi_{t}}\in \left\{1,2,\ldots ,c_{\varphi_{t}}\right\}$ and $c_{\varphi_{t}}=C_{\varphi_{t}}^{m_{k}}=\frac{m_{k}!}{\varphi_{t}!\left(m_{k}-\varphi_{t}\right)!}$.
$z_{\varphi_{t},n_{\varphi_{t}}}\left(k\right)$	A measurement cell specified by $\varphi_{t}$ and $n_{\varphi_{t}}$ at scan *k*.
$\chi_{k}^{t}$	The event that target *t* exists at scan *k*.
$\varepsilon_{j}$	The *j*th feasible joint event (FJE) which assigns measurement cells to tracks.
$\eta^{t}=z_{0}\left(k\right)$	The data association event for that no measurement cell is associated to track *t*.
$\eta^{t}=z_{\varphi_{t},n_{\varphi_{t}}}\left(k\right)$	The data association event for that measurement cell $z_{\varphi_{t},n_{\varphi_{t}}}\left(k\right)$ is associated to track *t*
